# Overtime working patterns and adverse events in work-related suicide cases: hierarchical cluster analysis of national compensation data in Japan (fiscal year 2015–2016)

**DOI:** 10.1007/s00420-021-01760-5

**Published:** 2021-09-25

**Authors:** Yuki Nishimura, Takashi Yamauchi, Takeshi Sasaki, Toru Yoshikawa, Masaya Takahashi

**Affiliations:** 1 Research Center for Overwork-Related Disorders, National Institute of Occupational Safety and Health, Japan Organization of Occupational Health and Safety, 6-21-1 Nagao, Tama-ku, Kawasaki, 214-8585 Japan; 2 Department of Public Health and Environmental Medicine, The Jikei University School of Medicine, Tokyo, Japan

**Keywords:** Working overtime, Karojisatsu, Karoshi, Mental health, Suicide, Japan, Cluster analysis

## Abstract

**Background:**

Although various work-related adverse events affect workers’ mental health, the association between long working hours and mental disorders remains unclear. We investigated the characteristics of overtime work and work-related adverse events among all cases of compensated work-related suicide in Japan to empirically reveal the context of the serious consequences.

**Methods:**

We analysed all 167 cases of mental disorders resulting in suicide that were compensated in fiscal year 2015–2016. Hierarchical clustering was applied to the overtime working history. Work-related adverse events were also evaluated as the qualitative aspects of their jobs.

**Results:**

More than half of the cases committed suicide within a month of developing a mental disorder. The Administrative and professional or engineering workers had a higher suicide rate. The clustering analysis revealed chronic long working hours (19%), gradual increase (27%), or rapid increase (25%) in working hours before the onset of a mental disorder. A group of cases with less overwork experienced more interpersonal conflicts.

**Conclusion:**

This is the first study to employ a clustering technique to objectively reveal the actual working patterns behind suicide. The patterns of working overtime before the onset of mental disorders varied considerably among the cases. Taking the transition of working overtime into account may provide clearer insight into the relationship between long working hours and workers’ mental health. These results highlight the need for countermeasures especially for causes of chronic overworking, drastic increases in working hours, and interpersonal conflicts to prevent work-related suicide.

**Supplementary Information:**

The online version contains supplementary material available at 10.1007/s00420-021-01760-5.

## Introduction

Although it has been a long time since Japan gained notoriety for disgracefully long working hours and a high suicide rate, overwork-related deaths, known as “Karoshi” (overwork-related death from cerebrovascular and cardiovascular diseases) and “Karojisatsu” (overwork-related suicide due to mental disorders), remain high in the country. The number of compensation claims for work-related mental disorders submitted to the Industrial Accident Compensation Insurance (IACI) continues to grow, possibly reflecting increased social attention (Yamauchi et al. 2017). Among the numerous issues, suicide is an irreversible and urgent issue that must be addressed to avoid loss of life.

While various types of work-related events such as disasters, interpersonal conflict in the workplace, and job characteristics (Tsuno et al. 2018; Pennington et al. 2018; Nishimura et al. 2020; Gerhardt et al. 2021) have been identified as risk factors for workers’ mental health, the association between long working hours and workers’ mental health is still contentious. During the IACI investigation for compensation determination, the existence and severity of 36 types of work-related events, including interpersonal conflict and overtime work, were evaluated as possible backgrounds for mental disorders (Nishimura et al. 2020). However, earlier studies, including meta-analyses and systematic reviews, have reported inconsistent relationships between working overtime and mental disorders. The first meta-analysis on the topic by Sparks et al. (1997) concluded that there were small but significant correlations between long working hours and mental health issues. Watanabe et al. (2016) reported that the effect of overtime work on mental health was small and not significant. Bannai and Tamakoshi (2014) defined long working hours as ≥ 40 h per week or ≥ 8 h per day to address the inconclusive associations between long working hours and mental disorders. They found significant adverse effects of long working hours on depression, anxiety, and sleep conditions. In addition, the most recent meta-analysis reported a significant relationship between long working hours and occupational health, including mental health, by summarising 243 reports published after the Spark’s article (Wong et al. 2019). According to the literature, these varying results were due to not only study design, lack of uniform confounders in the analysis, differences in cultural backgrounds, but also inconsistent definition of overtime work. Additionally, the lack of severe cases in their datasets may have also led to inconsistencies. Although long working hours are not directly related to suicide, depression is strongly related to suicide through suicide ideation and attempts (Wenzel and Beck 2008; Howard and Krannitz 2017). Investigating the reality of long working hours, related job characteristics and adverse events in workers may provide a new perspective.

The Research Center for Overwork-Related Disorders (RECORDS) at the National Institute of Occupational Safety and Health, Japan, has a nationwide database of all compensated IACI cases for mental disorders, including suicide following a mental disorder. Since the database covers all compensated cases according to national standards, a high coverage rate and reliability can be expected, especially in severe cases. By utilising data from the database, as a study found that people who committed suicide tended to experience longer working hours than patients who survived (Yamauchi et al. 2018). However, no earlier study has objectively classified the history of overtime working hours and discussed its background. Thus, we aimed to investigate the characteristics of Japanese work-related suicide cases in relation to long working hours and the possible background of such harsh working conditions by applying hierarchical clustering to the overtime working data extracted from the national database. Martin et al. (2019) used clustering to classify suicide cases of soldiers. However, no study has employed a clustering technique for the overtime hours of workers.

## Methods

### Data source and procedures

**Fig. 1:**
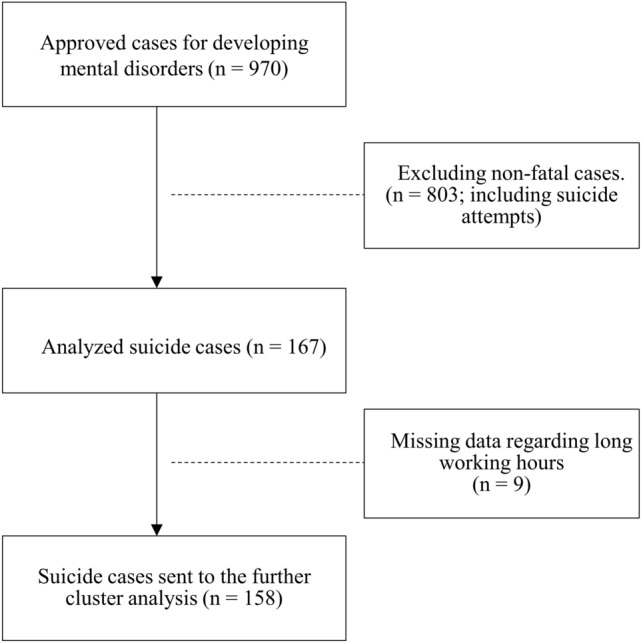
Flow chart for case selection

The database employed in the present study was developed by RECORDS at the National Institute of Occupational Safety and Health, Japan, with the support of the Ministry of Health, Labour and Welfare (MHLW) based on the act on Promotion of Preventive Measures against Karoshi and Other Overwork-Related Health Disorders. Of the 970 approved mental disorder cases between April 2015 and March 2017 (two years), which was the latest available dataset at the time, we extracted 167 suicide cases for analysis in the present study. Of these, nine cases were eliminated from subsequent cluster analysis due to incomplete data on long working hours (Fig. 1). Industrial and occupational classifications of suicide cases were conducted according to the Japan Standard Industrial Classification and the Japan Standard Occupational Classification established by the government. The work-related suicide occurrence ratio per one million workers in terms of industry and occupational classification was calculated based on the Labour Force Survey published by the Statistics Bureau, Ministry of Internal Affairs and Communications, Japan.

The authors assert that all procedures undertaken in this work comply with the ethical standards of the relevant national and institutional committees on human experimentation and with the Helsinki Declaration of 1975, as revised in 2008. Although the analysed database consisted solely of information on the deceased, all relatives of the suicide cases were informed of the study goals and had the opportunity to opt-out via the official website of the MHLW and RECORDS if they did not want their case to be used for research purposes (https://www.jniosh.johas.go.jp/rule/pdf/optout_overwork.pdf; in Japanese; Accessed 25th March 2021). All procedures including opt-out were approved by the Ethics Review Committee of the National Institute of Occupational Safety and Health, Japan (H3009 and 2019N20).

### Overtime working hours

Overtime working hours before the estimated onset of mental disorder were calculated during the IACI investigation utilising company records and any other available objective source such as the log of one’s personal computer and workplace security system. IACI defines overtime as working hours in excess of 40 h per week.

### Work-related adverse events

The presence or absence of adverse workplace events is examined by officers for all compensation requests submitted to the IACI according to the list contained in the 2011 Recognition Criteria for Occupational Mental Disorders. The list includes two extremely severe events (extremely psychologically stressful events and extremely long working hours) and 36 specific work-related adverse events. All cases identified as experiencing extremely severe events in this study were classified as extremely long overworking (≥ 160 h in 1 month prior to the onset of mental disorders or equivalent, such as > 120 h in three consecutive weeks prior to the onset of mental disorders), and no case was approved because of experiencing extremely psychologically stressful events (such as life-threatening injuries). The 36 work-related events were sorted into the following six groups: (1) experience of an accident or disaster; (2) failure in work, excessive responsibility, etc.; (3) quality and quantity of duties (such as overtime work for ≥ 80 h in a month and continuous work for ≥ 2 weeks without a break); (4) changes in roles and positions; (5) interpersonal conflicts; and (6) sexual harassment. Since two extremely severe events and 36 work-related adverse events are listed for the IACI review and not for research purposes, there is some overlap in the events. Therefore, we evaluated the experience of work-related adverse events by focusing on the six-event types which is the summary version of all events.

### Mental disorders

Being affected by a mental illness (Code:F mental and behavioural disorders of ICD-10) solely due to the psychological burden of work is one of the requirements to get compensated by IACI. Most of the cases included in the current study were approved for F3: mood (affective) disorders or F4: neurotic, stress-related, and somatoform disorders. Since applications of IACI are filed after the suicide completion, the IACI investigator employs medical records (if available) and testimony of family and co-workers to estimate the type of mental disorder and the onset. All suicide cases were also reviewed by a regional special committee composed of psychiatrists. Since the date of onset is uncertain for the above reasons, dates from onset to death are classified into five groups before analysis (Supplemental Table 1).

### Statistical analysis

All statistical analyses were conducted using R software version 3.6.3. Hierarchical clustering with Wald’s agglomeration method based on Euclidean distance was conducted on the overtime working data using the hclust function of R. The dendrogram was cut into four clusters by visual inspection and referring to the predefined criterion (≥ 30 cases per group) (can be found in the supplemental data). A mixed-design analysis of variance (mdANOVA) on long working hours with clusters (A to D) and months (one-six months prior to the onset) was conducted to verify the clustering results. Greenhouse–Geisser correction was applied where required by Mendoza’s multiple sphericity test. Fisher’s test for count data was performed to test the relationship between overtime working clusters and six types of work-related adverse events. The significance level of all statistical tests was set at *p* < 0.05.

## Results

### Demographic data

Of the167 cases, 97.0% (162) were men. The mean and standard deviation of age were 40.1 years (± 10.2) and 40.4 years (± 10.2) at the onset of mental disorder and suicide completion, respectively. Most cases (156, 93.4%) were approved as having “F3 mood [affective] disorders,” eight (4.8%) were “F4 neurotic, stress-related, and somatoform disorders” and three (1.8%) were “F2 Schizophrenia, schizotypal, and delusional disorders” based on the ICD-10 classification. In more than half of the cases (86 cases, 51.5%), the number of days from the onset of the mental disorder until suicide was < 30 days. Additionally, more than 70% (121 cases) committed suicide within < 90 days from the onset of the disease (Supplemental Table 1).

In terms of industry, the manufacturing industry had the highest number of cases, with a suicide rate of 1.62 cases per million workers. Sixteen cases were from the scientific and technical services industry, where the number of cases per million workers was 4.83, which was relatively high when compared to the other industries (Table 1). In terms of the type of occupation, administrative and managerial workers showed the second-highest number of cases (24 in two years) and the highest occurrence ratio (8.22 cases per million workers). Professional and engineering workers showed the highest number of cases (62) and the second-highest occurrence ratio (2.89 cases per million workers).

**Table 1:** Number of cases by industry and type of occupation

	*n*	%	/million workers
Industry			
Agriculture/forestry	1	0.6	0.93
Fisheries	1	0.6	6.25
Construction	29	17.4	3.47
Manufacturing	34	20.4	1.62
Electricity/gas/heat supply/water	3	1.8	5.08
Information/communication	12	7.2	3.01
Transport/postal services	14	8.4	2.13
Wholesale/retail trade	13	7.8	0.67
Finance/insurance	6	3.6	1.94
Real estate/leasing	4	2.4	1.84
Scientific/technical services	17	10.2	4.83
Accommodation/food service	5	3.0	0.76
Living-related/personal services/ amusement services	1	0.6	0.28
Education/learning support	3	1.8	0.36
Medical/health care/welfare	14	8.4	0.72
Compound services	2	1.2	1.67
Services/N.E.C	8	4.8	0.95
Occupational classification			
Administrative/managerial workers	25	15	8.22
Professional/engineering workers	67	40.1	2.89
Clerical workers	24	14.4	0.9
Sales workers	18	10.8	1.05
Service workers	6	3.6	0.31
Security workers	2	1.2	0.79
Agriculture/forestry/fishery workers	1	0.6	0.23
Manufacturing process workers	11	6.6	0.57
Transport/machine operation workers	3	1.8	0.69
Construction/mining workers	7	4.2	1.17
Carrying/cleaning/packaging/related workers	3	1.8	0.33
Total	167	100.0	1.62

Industry and occupational classification are classified according to Japan Standard Industrial Classification and Japan Standard Occupational Classification

### Clustering of history of overtime working

**Fig. 2:**
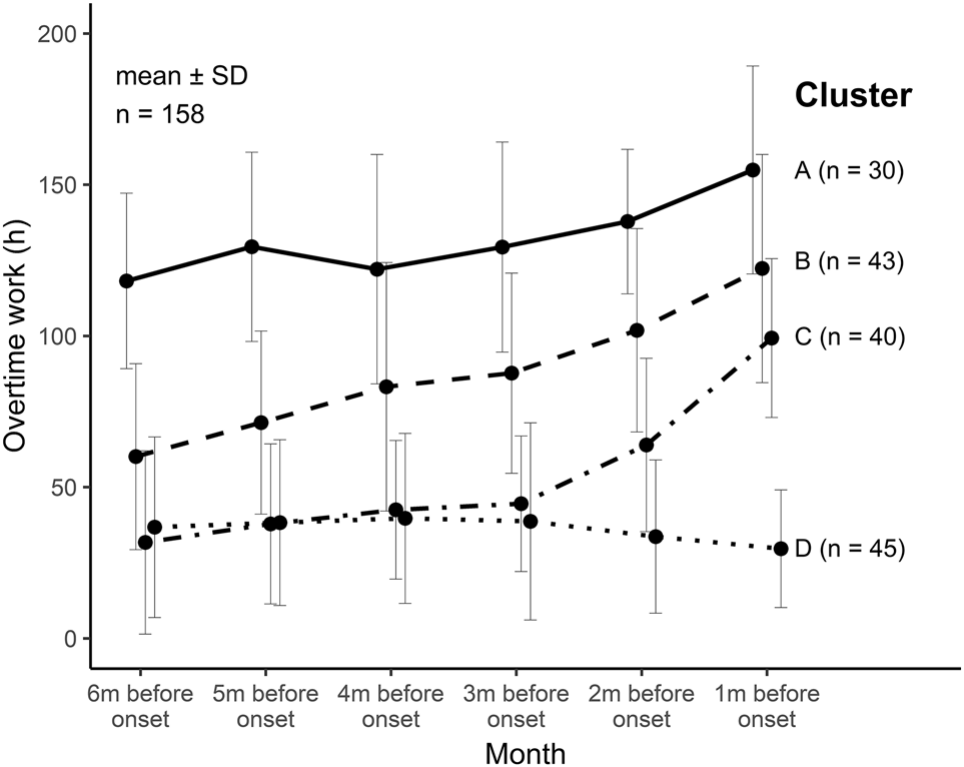
Mean overtime work before the onset of mental disorders according to clusters. Cluster A (chronic long-working group), Cluster B (chronic and gradual increase group), Cluster C (rapid increase group), and Cluster D (lower stable group) are indicated by a solid line, dashed line, dash-dotted line, and dotted line, respectively

Figure 2 shows the mean overtime hours of one to six months prior to the onset of mental disorder for each cluster (the cluster dendrogram can be found in the supplemental material). We named the four clusters based on their trends as follows: (A) chronic long-working group (30 cases), (B) chronic and gradual increase group (43 cases), (C) rapid increase group (40 cases), and (D) lower stable group (45 cases). The mean (SD) hours of overtime working 6 months prior to suicide was 132.0 (± 18.7), 87.7 (± 11.6), 53.3 (± 13.3), and 36.1 (± 19.8) hours for groups A to D, respectively.

The mdANOVA of the overtime hours revealed significant main effects of cluster [*F*(3) = 244.00, *p* < 0.001, *η*^2^_G_ = 0.58] and month [*F*(4.09) = 38.53, *p* < 0.001, *η*^2^_G_ = 0.15]. Moreover, the interaction between the two factors reached significance, indicating successful pattern classification of overtime hours [*F*(12.26) = 9.48, *p* < 0.001, *η*^2^_G_ = 0.12].

### Cross analysis

Table 2 shows the cross-tabulation of overtime work clusters, and industry or type of occupation. Although statistical tests were not performed due to an insufficient number of cases, administrative and managerial workers tended to be classified in cluster A or B, suggesting extremely long working hours in these job types. Conversely, professional and engineering workers tended to experience a gradual increase in overtime work prior to onset.

**Table 2:** Cross analysis of industry or occupational classification and the cluster

	Cluster^§^			
	A	B	C	D
Industry				
Agriculture/forestry	0	1	0	0
Fisheries	0	0	1	0
Construction	7	10	4	7
Manufacturing	11	3	5	13
Electricity/gas/heat supply/ water	0	1	1	1
Information/communication	1	3	6	2
Transport/postal services	5	4	3	2
Wholesale/retail trade	1	4	3	5
Finance/insurance	0	1	2	3
Real estate/leasing	0	3	1	0
Scientific/technical services	1	5	6	4
Accommodation/food service	1	3	0	1
Living-related/personal services/amusement services	0	1	0	0
Education/learning support	0	0	2	0
Medical/health care/welfare	1	3	1	6
Compound services	0	0	2	0
Services/N.E.C	2	1	3	1
Occupational classification				
Administrative and managerial workers	8	10	3	3
Professional and engineering workers	11	13	17	21
Clerical workers	2	4	8	9
Sales workers	2	5	5	6
Service workers	0	5	0	0
Security workers	0	0	2	0
Agriculture, forestry, and fishery workers	0	0	1	0
Manufacturing process workers	4	1	1	4
Transport and machine operation workers	0	1	2	0
Construction and mining workers	1	4	1	1
Carrying, cleaning, packaging, and related workers	2	0	0	1
Total *n* of clusters	30	43	40	45

^§^Overtime working clusters in 6 months before the onset of mental disorder

Table 3 shows the number of cases classified according to the six work-related event types for each cluster. Fisher’s tests performed for each event type showed a significant relationship between the type of long working hours and, excessive workload (*p* = 0.011; *n* = 158) and interpersonal conflicts (*p* = 0.017; *n* = 158). Since extremely long working hours in the special events are defined as ≥ 160 h of overwork in a month prior to the onset or equivalent, a significant relationship with this cluster is a natural consequence. Those who experienced human relationship issues were less likely to work longer than those who experienced other events.

**Table 3:** Cross analysis of work-related adverse event and cluster

	Cluster^§§^				
Event type	A (*n* = 30)	B (*n* = 43)	C (*n* = 40)	D (*n* = 45)	*p* value^§^
(1) Accident or disaster	0	1	0	4	0.097
(2) Failure in work, excessive responsibility	7	17	18	16	0.297
(3) Excessive workload	13	30	31	24	0.011*
(4) Position change	7	10	11	16	0.569
(5) Interpersonal conflicts	4	11	12	21	0.017*
(6) Sexual harassment	0	0	0	0	Not tested

The sum of number of cases attributed to each cluster is not necessarily equal to the absolute number of cases for each cluster since many cases are compensated by experiencing multiple types of events

*p < 0.05

^§^*p* values of Fisher’s exact test operated at each event type between clusters

^§§^Overtime working clusters in 6 months before onset of mental disorder

## Discussion

This study aimed to characterise work-related suicide cases approved for compensation in light of overtime work and work-related adverse events 6 months prior to the onset of mental disorders. This is the first study to objectively reveal patterns of overtime working history over 6 months.

### General characteristics

Nearly all cases were men, and they were approximately 40 years old when they committed suicide. To begin with, the suicide rate is higher among men than women in most countries, including Japan (Turecki et al. 2019; Kino et al. 2019). However, our findings showed a greater gender imbalance than that. The strong gender imbalance can be due to the subject’s career stages, bias in an application for compensation, and the Japanese situation of co-participation of all genders in the workplace. With regard to work life, responsibility and workload increase around the age of 40 years. This could possibly have led to an excessive workload and mental pressure. The application of IACI compensation requires significant effort. Considering that many families in Japan depend on men for their main source of income, suicide committed by men directly impacts the family’s economic situation. This may introduce a higher rate of compensation application by the widow to protect her family’s daily lives. Although the situation concerning gender equality in the workplace is improving, women are still less likely than men to hold administrative positions, which may have also led to the gender imbalance in the present study.

Despite the severity or duration of depression, the first 3 months after the onset of a major depressive episode are those with the highest risk of suicide attempts in individuals vulnerable to suicidal behaviour (Kawahito et al. 2012; Randall et al. 2014). Although detailed information regarding the development of mental disorders is lacking in the RECORDS database, the number of days from the onset of a depressive disorder estimated from observed depressive episodes until suicide was < 90 days in many of the analysed cases. Quicker intervention, especially for high-risk workers with known risk factors such as insomnia, feelings of oppression, or history of suicide attempts may be beneficial in preventing suicide (Turecki and Brent 2016; Steele et al. 2018; Turecki et al. 2019).

### Industry and type of occupation

In terms of industry, the tertiary industry, which predominantly includes office work, was the most frequent in terms of cases. Regarding occupation, white-collar workers such as administrative workers showed a high rate of work-related suicide. Sato et al. (2020) reported that the mental health effects of long working hours differ between white-collar and blue-collar workers due to differences in work styles and expectations. They reported a significant association between deteriorated mental health, long working hours and weekend work. Myrtek et al. (1999) reported inconsistent results between subjective and objective measures of mental strain. Higher levels of mental strain (predicted from heart rate variability) were observed in blue-collar workers, while white-collar workers reported higher levels of subjective stress at work (and outside work as well). Taking the results of cross analysis with patterns of overtime work into account, an insufficient degree of job control and other social factors with increasing working time may increase the risk of suicide among white-collar workers (Tsuno et al. 2019).

### Patterns of overtime work

The hierarchical clustering showed variation in overtime work and its relationship with industry, type of occupation, and work-related adverse events. The time series of long working hours was not uncovered for a long time because of the low accessibility to relevant data. We employed national data to reveal the reality of long working hours before the onset of a mental disorder that led to suicide.

Our results showed that many victims worked very longer than normal schedule. As discussed earlier, there have been many inconsistent reports on the association between long working hours and mental disorders (Sparks et al. 1997; van der Hulst 2003; Bannai and Tamakoshi 2014; Watanabe et al. 2016). The reviewed literature could not detect an association between working hours and severe consequences due to the criteria used to define long working hours. The frequently employed criteria of long working hours in the literature was ≥ 40 h per week compared with, for example, group A workers in the present study who attended work for ≥ 70 h per week. When the rest periods are short, sleep duration and quality decrease (Ikeda et al. 2018). Since many victims experience extremely long working hours that lead to shorter daily rest periods, they might have been experiencing difficulty in recovering from physiological and psychological fatigue due to a lack of sufficient daily sleep (Caldwell et al. 2019). Thus, even if long hours of work themselves are not a cause of depression, they may still be an important risk indicator of depression and suicide among workers.

Changes in overtime work may also have exerted a psychological effect on mental health. Since changes in the quantity and quality of work affect mental health status (Nishimura et al. 2020), the observed increase in overtime work in groups B and C might severely impact the victim’s mental health along with the cause of the extended working hours. Moreover, for white-collar workers, working on weekends has a one-and-a-half-to-two-fold increase in negative risks compared to those working overtime on weekdays in the case of mental ill-health (Sato et al. 2020). In the present study, some cases were approved for working for more than > 14 consecutive days. With a higher rate of suicide among white-collar workers, investigating the cause of increased working hours and securing weekends would be effective in improving the current situation.

As shown in Table 2, administrative and managerial workers were more likely to experience chronic overtime working. The working hours for professional and engineering workers were likely to increase before the onset of mental disorders, indicating the occurrence of work-related events that augment their workload. On the other hand, the existence of other stressors besides excessive workload, leading to the development of mental disorders was also implied. Further investigations with a larger number of cases are required in the future. Group D members were more likely to have human relationship events than the other groups (Table 3). Interpersonal conflicts are a widely known risk factor for mental disorders (Ikeda et al. 2009; Inoue and Kawakami 2010). In sum, even if working hours and patterns are not good indicators of suicide risk, focusing on its patterns might help to understand the situation that workers face.

### Limitations

The present study included all cases approved for compensation by the IACI for committing suicide following the work-related development of a mental disorder. All applications were investigated by dedicated officers and approved according to the national criteria. However, a sampling bias may still exist. First, IACI only covers people working for others and not those who are self-employed or civil servants (civil servants are covered by other programmes). Second, not all bereaved families may apply for IACI compensation. Finally, there are some unapproved cases due to insufficient evidence of work-related adverse events. These were not included in the present study, and thus, it must be noted that the current data are likely to represent more severe cases in Japan. Investigation of suicide cases is conducted after the suicide, thus missing the testimony of the deceased individual. Therefore, the contribution of mental load, work-related events, and, particularly, diseases is uncertain. The hierarchical analysis method employed in this study forcibly classifies the input data into several groups based on the relative relationship of the data. Thus, our results show the tendency of the focused cases within the targeted years and are not representative of all suicide cases in Japan.

## Conclusions

The present study revealed major patterns of overtime work such as chronic overtime work and rapid increase of overtime work prior to the onset of mental disorder in Japanese work-related suicide cases and its possible backgrounds by utilising the national database and the objective method. Taking the transition of overtime working prior to suicide attempt and onset of mental disorder into account may provide clearer insight into the relationship between long working hours and workers’ health in future studies. The possible burden of various patterns of transition in working hours on workers should be investigated in the future. Although the current study consists of suicide cases only, the findings can contribute to reducing victims of overtime work. First, those who are engaged in chronic long working hours need weekends by identifying the cause of the long hours. When working hours increase in consecutive months, the prevention of sudden events, such as accidents and incidents that extend the working hours, is critical to free workers from their psychological loads. As discussed earlier, not long working hours, but the cause behind the overtime working will be the key problems to be solved to prevent work-related depression and suicide. Investigating the possible subsequent changes in working hours of various types of work-related events may also contribute to appropriate labour management.

## Supplementary Information

Below is the link to the electronic supplementary material.

Supplementary file1 (DOCX 249 KB)

## Data Availability

The dataset of suicide cases used and/or analysed during the current study are not publicly available since individual privacy could be compromised by combining information in the dataset.

## Abbreviations

IACI: Industrial accident compensation insurance
mdANOVA: Mixed-design analysis of variance
MHLW: Japanese Ministry of Health, Labour and Welfare
RECORDS: Research Center for Overwork-Related Disorders

## Code availability

Codes used during the current study are available from the corresponding author on reasonable request.
