# Supplementary material for: Overtime working patterns and adverse events in work-related suicide cases: hierarchical cluster analysis of national compensation data in Japan (fiscal year 2015–2016)
Source: Int Arch Occup Environ Health. 2021 Sep 25;95(4):887–95. doi: 10.1007/s00420-021-01760-5 (PMC9038865; doi:10.1007/s00420-021-01760-5)
Supplement: Supplementary file 1 — Supplementary file1 (DOCX 249 KB) [file 420_2021_1760_MOESM1_ESM.docx]

# Supplementary materials


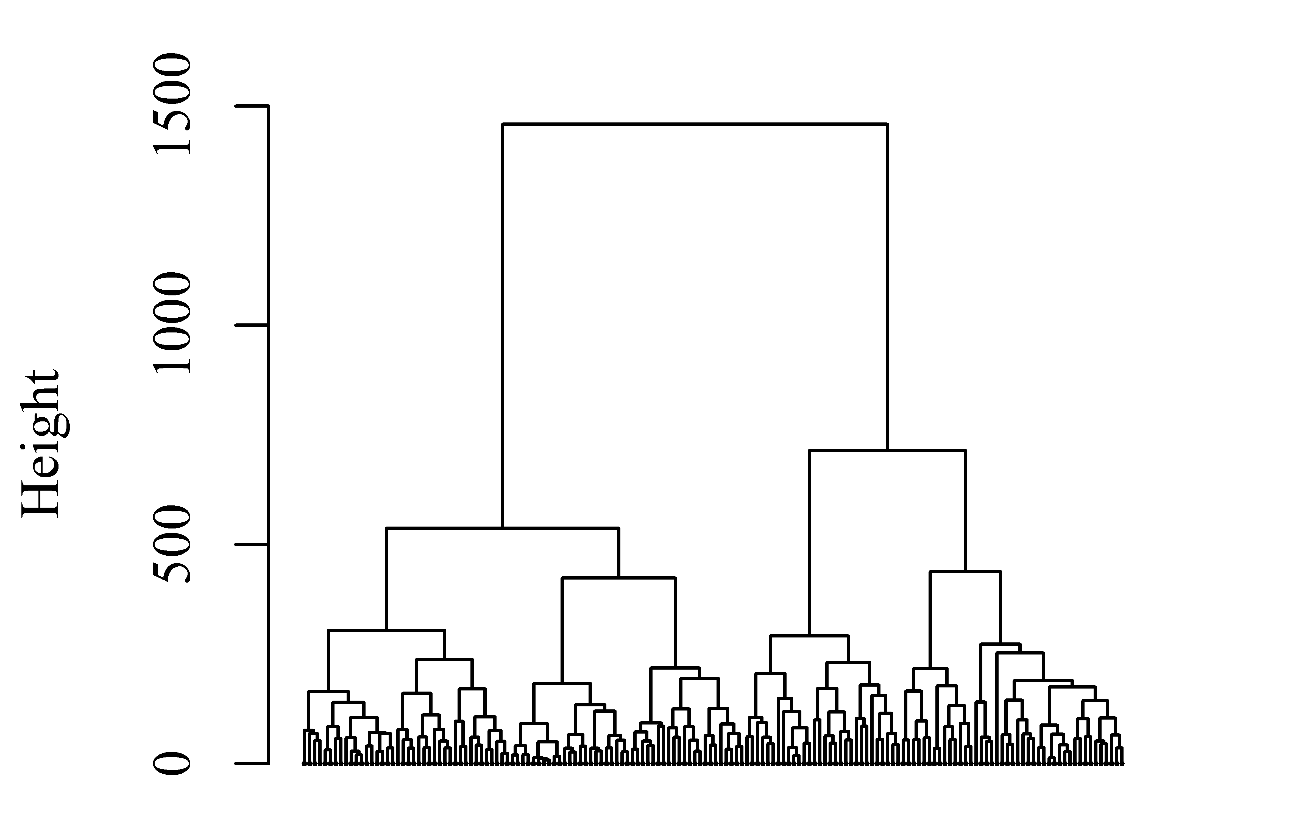


Cutting line

**Supplemental Fig. 1** Cluster dendrogram. Dashed line indicates the cutting line of the clusters.

| **Supplemental Table 1** Days until suicide after onset of the mental disorders | | |
| --- | --- | --- |
|  | n | % |
| **Days** |  |  |
| < 30 | 86 | 51.5 |
| 30-89 | 35 | 21.0 |
| 90-179 | 17 | 10.2 |
| 180-359 | 10 | 6.0 |
| 360- | 19 | 11.4 |
| **Total** | 167 | 100.0 |
